# Characterization of intra- and inter-host norovirus P2 genetic variability in linked individuals by amplicon sequencing

**DOI:** 10.1371/journal.pone.0201850

**Published:** 2018-08-09

**Authors:** Aurora Sabrià, Rosa M. Pintó, Albert Bosch, Josep Quer, Damir Garcia-Cehic, Josep Gregori, Angela Dominguez, Mónica Carol, Maria-Rosa Sala-Farré, Susana Guix, the Working Group for the Study of Outbreaks of Acute Gastroenteritis in Catalonia

**Affiliations:** 1 Enteric Virus Laboratory, Department of Genetics, Microbiology and Statistics, University of Barcelona, Barcelona, Spain; 2 Institute of Nutrition and Food Safety (INSA·UB), University of Barcelona, Barcelona, Spain; 3 Liver Unit, Internal Medicine, Laboratori de Malalties Hepàtiques, Vall d’Hebron Institut Recerca-Hospital Universitari Vall d’Hebron (VHIR-HUVH), Autonomous University of Barcelona, Barcelona, Spain; 4 Centro de Investigación Biomédica en Red (CIBER) de Enfermedades Hepáticas y Digestivas (CIBERehd), Instituto de Salud Carlos III, Madrid, Spain; 5 Roche Diagnostics S.L., Sant Cugat del Vallès, Spain; 6 CIBER Epidemiología y Salud Pública (CIBERESP), Instituto de Salud Carlos III, Madrid, Spain; 7 Department of Public Health, University of Barcelona, Barcelona, Spain; 8 Public Health Agency of Catalonia, Generalitat de Catalunya, Barcelona, Spain; Centers for Disease Control and Prevention, UNITED STATES

## Abstract

Noroviruses are the main cause of epidemics of acute gastroenteritis at a global scale. Although chronically infected immunocompromised individuals are regarded as potential reservoirs for the emergence of new viral variants, viral quasispecies distribution and evolution patterns in acute symptomatic and asymptomatic infections has not been extensively studied. Amplicons of 450 nts from the P2 coding capsid domain were studied using next-generation sequencing (454/GS-Junior) platform. Inter-host diversity between symptomatic and asymptomatic acutely infected individuals linked to the same outbreak as well as their viral intra-host diversity over time were characterized. With an average of 2848 reads per sample and a cutoff frequency of 0.1%, minor variant haplotypes were detected in 5 out of 8 specimens. Transmitted variants could not be confirmed in all infected individuals in one outbreak. The observed initial intra-host viral diversity in asymptomatically infected subjects was higher than in symptomatic ones. Viral quasispecies evolution over time within individuals was host-specific, with an average of 2.8 nt changes per day (0.0062 changes per nucleotide per day) in a given symptomatic case. Nucleotide polymorphisms were detected in 28 out of 450 analyzed nucleotide positions, 32.14% of which were synonymous and 67.86% were non-synonymous. Most observed amino acid changes emerged at or near blockade epitopes A, B, D and E. Our results suggest that acutely infected individuals, even in the absence of symptoms, which go underreported and may enhance transmission, may contribute to norovirus genetic variability and evolution.

## Introduction

Human Norovirus (HuNoV) is the dominant agent involved in acute gastroenteritis causing one fifth of all cases worldwide, and a major cause of foodborne illness [1]. It is highly infectious [2] and is transmitted by the fecal-oral route, either through contact with infected individuals or through exposure to contaminated food and water, although several other modalities have been described such as via aerosolized viral particles in vomitus [3]. It is responsible for 685 million cases every year affecting both, developed and undeveloped countries. In developed countries, it is estimated that every year HuNoVs cost $60 billion due to health care costs like complications and productivity losses due to absenteeism [4].

The viral RNA genome is about 7500 nt long with three open reading frames (ORFs). The ORF1 encodes for a polyprotein that is processed co- and post-translationally by the viral protease to generate six mature active proteins [5]. ORF2 and ORF3 are translated from subgenomic RNA and encode VP1 and VP2, respectively. VP1 is the major capsid protein and is organized into two domains [6], the shell (S) and the protruding (P) domain. P domain is subdivided into two subdomains P1 and P2. P1 subdomain is more conserved than P2 which is the most exposed region of the capsid and contains the Human Histo-Blood Group Antigen (HBGA) binding function and antigenic determinants [7].

Noroviruses are classified into seven genogroups (GI to GVII) and nearly 40 different genotypes, being genogroup I and II the most relevant for human infection [8]. Genogroup II is the most prevalent one, accounting for around 90% of all infections, and GII.4 has been the most commonly isolated genotype in most parts of the world during the last decade [9,10]. New genetic clusters of GII.4 arise every 2 to 4 years under the pressure of herd immunity [11,12], although the reservoir for such variants is still poorly characterized. In individuals chronically infected with HuNoV (mostly immunocompromised patients) new variants may arise due to the accumulation of mutations during the protracted virus replication. In addition, a lower replication fidelity of GII.4 pandemic strains has also been related to an increased intra-host diversity [12,13,14,15,16].

However, despite it is clear that HuNoV intra-host population diversity is higher in persistently infected immunocompromised individuals, HuNoV quasispecies distribution and evolution patterns in both symptomatic and asymptomatic acute infections has not been extensively studied [12]. Genetic variability is one of the main factors contributing to the high incidence of HuNoV in the human population, and a better knowledge of HuNoV evolution driving forces is of clinical and public health interest.

The aim of this study was to analyze the inter-host HuNoV GII.4 diversity between symptomatic and asymptomatic infected individuals linked to the same outbreak, as well as their viral intra-host diversity over time, using amplicon next-generation sequencing (NGS) approaches.

## Material and methods

### Sample collection

Fecal samples and epidemiological data (outbreak setting, type of transmission, clinical symptoms and time of sample collection) were collected from food-handlers and health-care workers of one foodborne outbreak that took place in a restaurant (RCC11/10), and one person-to-person transmission outbreak that affected a nursing home (UVEVV51/10) (Table 1). Day 1 was defined as the first day of sample collection and not the first day of disease onset. For symptomatic individuals, first samples were taken during the first 3 days after the onset of symptoms. A serial stool sample was collected several days later from two individuals with symptomatic infection. Genotype and virus load for all samples were determined in two previous studies [17,18]. All studied samples (n = 8) were GII.4 New Orleans 2009. The study was approved by the Institutional Review Board (IRB00003099) of the Ethics Committee of the University of Barcelona. Written consent was not required because data were anonymized prior to analysis and gathered for outbreak control and public health purposes.

**Table 1:** Selected samples from food-handlers (FH) and health care workers (HCW) involved in outbreaks under study analyzed by NGS.

Outbreak	Setting	Type of transmission	Date of outbreak	Duration of outbreak (days)	Number of affected individuals/Attack rate (%)	Average age of affected individuals (years)	Subject	Patient description, symptoms, age (years)	Date of specimen collection	Speci-men	Viral load (genome copies/g stool)	Reads (22587 total)	Number of Haplotypes
RCC 11/10	Restaurant	Foodborne (shellfish, mussels)	18/5/10	4	82/50.3	42.2	A	FH, ill, 43	20/5/10	A-1	8,09x10^9^	2700	1
									28/5/10	A-9	1,33x10^9^	1795	1
							B	FH, not ill, 36	20/5/10	B-1	7,11x10^9^	5038	7
UVEVV 51/10	Nursing home	Person-to-person	10/11/10	12	21/21	75.1	C	HCW, ill, 68	17/11/10	C-1	2,07x10^8^	1275	2
							D	HCW, ill, 50	17/11/10	D-1	1,32x10^11^	4424	3
									22/11/10	D-6	3,37x10^10^	3653	18
							E	FH, not ill, 47	17/11/10	E-1	6,98x10^7^	2249	1
							F	FH+HCW, not ill	26/11/10	F-1	1,62x10^6^	1453	6

### RT-PCR

Viral RNA was extracted from 150 μl of 10% stool suspension in phosphate-buffered saline (pH 7.4) and was stored at −80°C. A two-step reverse transcription-PCR (RT-PCR) was employed to amplify a 450-bp region of the HuNoV genome encoding for P2. Expand Reverse Transcriptase (Roche Applied Science, Basel, Switzerland) was used for the RT and high-fidelity *Pwo* DNA Polymerase (Roche Applied Science, Basel, Switzerland) for the PCR. Primers are described in Table 2 and include a template-specific sequence and a universal M13 sequence required in the multiplex Ultra-Deep Pyro Sequencing (UDPS) reaction [19]. RT-PCR products were purified from agarose gel bands using the NucleoSpin Gel and PCR Clean-up kit (Macherey Nagel, Düren, Germany) and quantified using a NanoDrop1000 spectrophotometer (Thermo Scientific, Wilmington, DE).

**Table 2:** Primers used for the study.

Primer	Sequence (5’-3’)	Sense	Position^a^	Genome Region
13uNoV5956	GTTGTAAAACGACGGCCAGTGTCAACATCTGCACCTTCAGA^b^	Forward	5925–5945	P2
13dNoV6447	CACAGGAAACAGCTATGACCTACCCRCTGCATCCGGGCAT^b^	Reverse	6396–6415	P1

^a^ GenBank accession number GU445325

^b^ Underlined nt correspond to M13 forward and reverse primers, respectively

### Ultra-Deep Pyro Sequencing (UDPS)

UDPS was performed using a 454/GS-Junior deep sequencing platform (Roche, Branford, CT, USA). Briefly, after purified RT-PCR products were pooled equimolarly at 10^9^ molecules/ul, they were subjected to 15 cycles of re-amplification for the addition of the MIDs, multiplex identifier oligomers of 10bp. Amplification products were purified using the QIAquick gel extraction kit (Qiagen, Valencia, CA, USA), evaluated using the BioAnalyzer DNA 1000 LabChip (Agilent, Santa Clara, CA, USA), and quantified by PicoGreen assay (Invitrogen, Carlsbad, CA, USA). Massive parallel sequencing was performed in a 454/GS-Junior platform (Roche, Branford, CT, USA), using titanium chemistry (GS Junior+ Series, XL+ Kit), which enables sequencing up to 700-nt fragments.

Computations were made as previously described [20,21,22]. Briefly, the fasta file from the GS-Junior was demultiplexed to obtain a fasta file for each sample and strand. Reads not identified by MID and/or primer were discarded. The allowances were two mismatches in the specific sequence, three on the universal M13 sequence and one on the MID sequence with no indels. Sequences not covering the full amplicon or showing more than 2 Ns or 3 gaps were discarded. Sequences not observed on the forward and reverse strands at a minimum abundance of 0.1% were discarded. Different haplotypes, defined as reads covering the full amplicon, were identified and their frequencies computed as the number of observed reads. All sequence data are available from the SRA database (accession number SRP150980).

### Phylogenetic analysis

Phylogenetic analysis was performed using the UPGMA method (distance calculation by number of differences method, pairwise deletion) implemented in the MEGA7 program [23], and results were validated by 1000 bootstrap replicates.

## Results

### Relation between viral titer and number of reads

**Fig 1:**
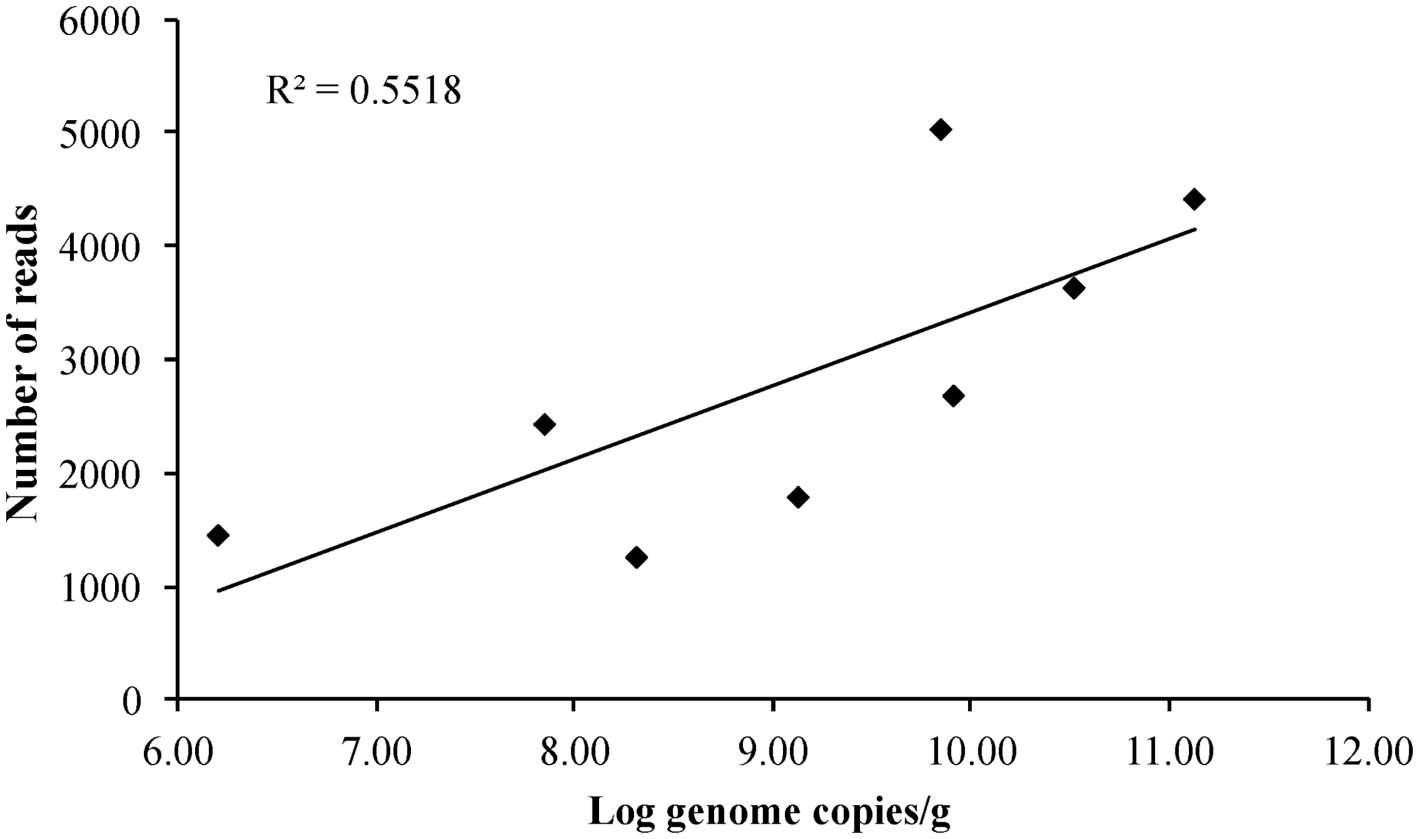
Correlation between viral titer in stool (log genome copies/g) and number of reads.

NGS was performed on 450-bp P2 amplicons on 8 selected samples. An average of 2848 reads per sample were obtained (range 1275–5038). Whole genome sequence analyses of other RNA viruses have demonstrated that the depth of coverage improves by increasing viral load [19]. As shown in Fig 1, we observed a moderate correlation (R^2^ = 0.552; p = 0.0347) between viral load and number of reads. In general, samples with highest viral load exhibited higher number of reads, while samples with lower titer yielded less reads. No correlation was found between viral load and number of haplotypes (R^2^ = 0.070), neither between number of reads and number of haplotypes (R^2^ = 0.143).

### Inter- and intra-host diversity between outbreak-linked symptomatic and asymptomatic cases

**Fig 2:**
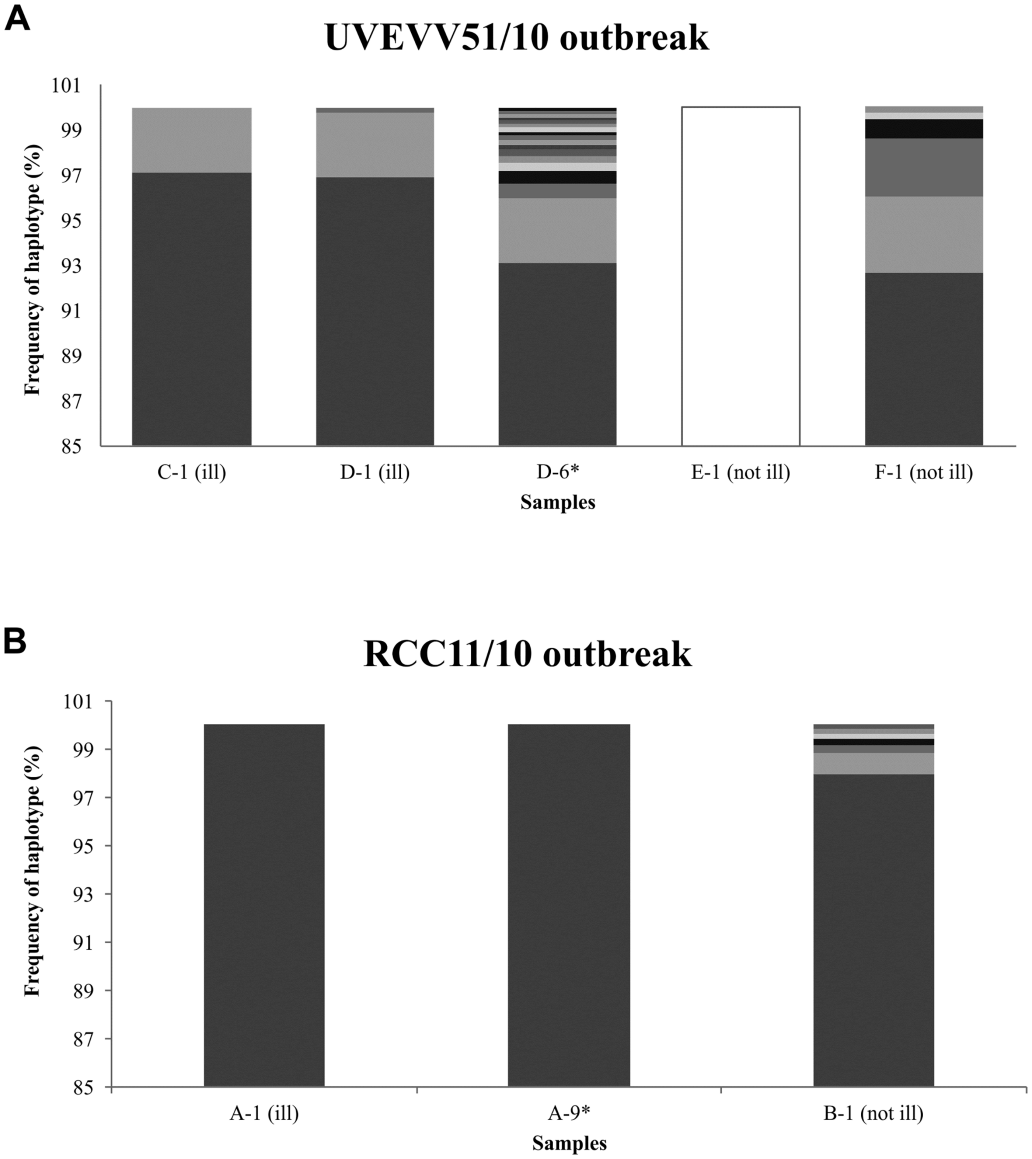
Comparison of the intra-host distribution of HuNoV haplotypes. Each unique haplotype is represented by alternate gray shading. **(A)** Subjects from RCC11/10 outbreak. **(B)** Subjects from UVEVV51/10 outbreak. Asterisk indicate serial sample.

**Fig 3:**
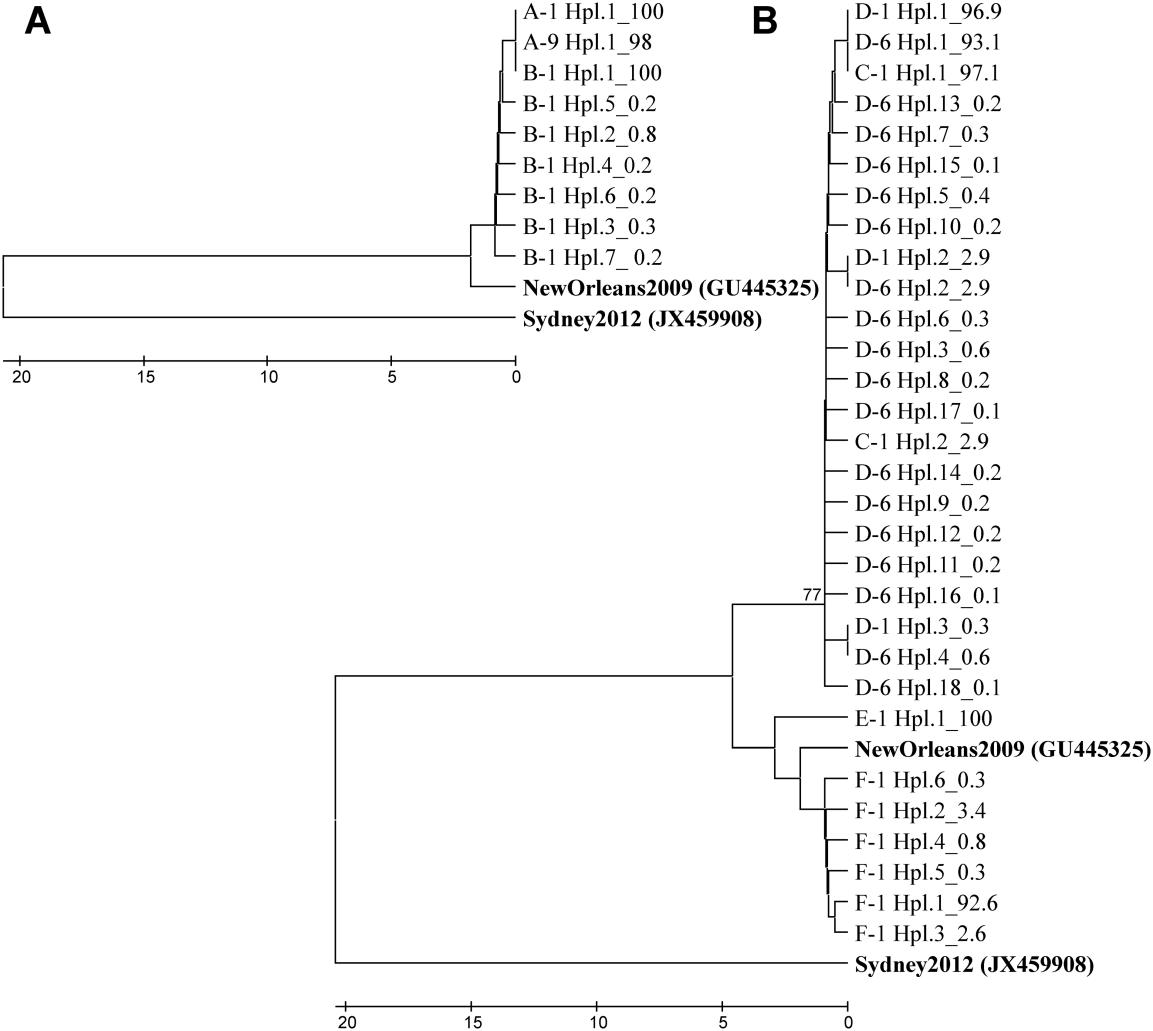
Phylogenetic analysis of haplotypes from RCC11/10 (A) and UVEVV51/10 (B) outbreak based on 450 bp of the P2 region. The nucleotide dendrogram was inferred using the UPGMA method with distance calculation by number of differences method using the MEGA7 software. A bootstrap of 1000 replicates was performed, and values above 75 are shown. Bold indicates reference strains. Haplotypes names are composed of sample name, number of haplotype.

Samples from two subjects where analyzed in RCC11/10 foodborne outbreak (subjects A and B). The single haplotype found in subject A was also the most common haplotype occurring in subject B (97.98%), who was infected from the same source but who did not show symptoms (Fig 2A). Of the other 6 minor haplotypes observed in subject B, 4 of them presented an amino acid change at evolving sites: P313L, I330T, T394I and P427L (Table 3 and Fig 3A).

**Table 3:** Amino acid differences between the haplotypes detected in RCC11/10 outbreak with reference to one of the samples within the outbreak. GII.4 reference strains are also provided at the top of the table.

	VP1 Amino acid Position					
	P2					P1
	Epitope A			Epitope D	Epitope E	
	294	313	330	394	413	427
2009 New Orleans (GU445325)	P	P	I	T	I	P
2012 Sydney (JX459908)	T	P	I	T	T	P
A-1 Hpl.1_100%	P	P	I	T	I	P
A-9 Hpl.1_100%						
B-1 Hpl.1_98.0%						
B-1 Hpl.2_0.8%						
B-1 Hpl.3_0.3%						L
B-1 Hpl.4_0.2%						
B-1 Hpl.5_0.2%		L				
B-1 Hpl.6_0.2%			T			
B-1 Hpl.7_0.2%				I		

Five samples from four patients were analyzed from the UVEVV51/10 outbreak. The data revealed marked differences between symptomatic and asymptomatic subjects. While symptomatic subjects C and D shared the most abundant haplotype, neither this haplotype nor any of the minor variants detected in these individuals were found in asymptomatic subjects E and F (Figs 2B and 3B). While all 5 haplotypes identified in the first specimen for subjects C and D only differed in synonymous changes, the single haplotype found in subject E, and the most abundant haplotype found in subject F, both asymptomatically-infected, differed in 2–3 amino acid positions (Table 4). Genetic diversity was especially notable in asymptomatic subject F, with 4 out of the 6 identified haplotypes containing several amino acid changes (Table 4). These results suggest either multiple sources of infection or a high degree of virus evolution within the selected infected individuals. Compared to GII.4 New Orleans reference strain, all haplotypes identified in subjects C and D differed in 2 common amino acids (P294S and I413T), and all but one also differed at an additional site (R339K) (Table 4). Haplotypes found in subjects E and F were more closely related to GII.4 New Orleans reference strain (Fig 3B), and only the haplotype identified in subject E showed P294S and R339K substitutions. All observed non-synonymous changes had been previously described [14,24,25].

**Table 4:** Amino acid differences between the haplotypes detected in UVEVV51/10 outbreak with reference to one of the samples within the outbreak. GII.4 reference strains are also provided at the top of the table.

	VP1 Amino acid Position																
	P2												P1				
			Ep. A								Ep. E						
	289	293	294	314	339	356	358	380	399	404	407	413	416	426	433	434	436
2009 New Orleans (GU445325)	D	I	P	T	R	A	F	N	E	V	S	I	V	F	F	F	S
2012 Sydney (JX459908)	D	I	T	T	R	A	F	N	E	V	S	T	V	F	F	F	S
C-1 Hpl.1_97.1%	D	I	S	T	K	A	F	N	E	V	S	T	V	F	F	F	S
C-1 Hpl.2_2.9%																	
D-1 Hpl.1_96.9%																	
D-1 Hpl.2_2.9%																	
D-1 Hpl.3_0.3%																	
D-6 Hpl.1_93.1%																	
D-6 Hpl.2_2.9%																	
D-6 Hpl.3_0.6%							L										
D-6 Hpl.4_0.6%																	
D-6 Hpl.5_0.4%				A													
D-6 Hpl.6_0.3%																	
D-6 Hpl.7_0.3%																	
D-6 Hpl.8_0.2%		T															
D-6 Hpl.9_0.2%								D									
D-6 Hpl.10_0.2%																	
D-6 Hpl.11_0.2%															L		
D-6 Hpl.12_0.2%																L	
D-6 Hpl.13_0.2%													A				
D-6 Hpl.14_0.2%									G								
D-6 Hpl.15_0.1%																	
D-6 Hpl.16_0.1%											I						
D-6 Hpl.17_0.1%					R												
D-6 Hpl.18_0.1%	G																
E-1 Hpl.1_100%												I					
F-1 Hpl.1_92.6%			P		R							I					
F-1 Hpl.2_3.4%			P		R							I					
F-1_Hpl.3_2.6%			P		R	V						I					
F-1_Hpl.4_0.8%			P		R							I		S			
F-1_Hpl.5_0.3%			P		R					M		I					
F-1_Hpl.6_0.3%			P		R							I					P

Overall, the intra-host viral diversity in asymptomatically infected subjects (B, E and F) at day 1 was higher than in symptomatic patients (A, C and D). Although differences were not statistically significant, the initial average of haplotypes per subject was of 4.7 in asymptomatic subjects versus 2.0 in symptomatic ones.

### Intra-host evolution diversity

While the intra-host viral diversity initially observed in asymptomatically infected subjects (B, E and F) was higher than in symptomatic patients (A, C and D), diversity evolution over time for the 2 individuals with a serial sample (A and D) showed markedly different trends. While in subject A, who was 43 years old, the single initial haplotype remained stable over the 8 days of infection, in subject D, a 50 year-old woman, HuNoV population diversity increased from 3 to 18 haplotypes in only 5 days (Table 1). Of note, this subject was also the one with the highest viral load. The phylogenetic analysis showed that of the 3 initial haplotypes in D-1, the most abundant haplotype slightly decreased from 96.9% to 93.1%, the second haplotype remained at a stable frequency of 2.9% and the third haplotype slightly increased from 0.3% to 0.6% (Fig 3B). During the following 5 days, variation occurred in 14/450 nt positions, which represents an average of 2.8 nt changes per day and 0.0062 changes per nucleotide per day. At the amino acid level, all 3 initial haplotypes detected in subject D were synonymous, and 11 out of the 18 evolved haplotypes contained amino acid changes (61.1%) at previously identified informative sites: D289G, I293T, T314A, F358L, N380D, E399G, S407I, V416A, F433L, and F434L (Table 4).

### Quasispecies analysis

Among all sequence variants isolated from the quasispecies from the 4 subjects that showed variation in the studied region (B, C, D, and F), 29 out of 450 analyzed nucleotide positions were variable (6.4%). Of these single nucleotide polymorphisms (SNPs), 34.5% were synonymous and 65.5% were non-synonymous.

Of note, 96.5% of these substitutions were transitions (58.1% of the transitions were substitutions U-to-C, 19.4% were A-to-G), and only 3.5% were transversions (G-to-U) (Table 5). Since HuNoV mutants with a high proportion of U-to-C and A-to-G transitions have been described in clinical samples and double-stranded RNA-specific adenosine deaminases (ADAR)-mediated editing of the viral RNA was suggested as the molecular mechanism responsible for them [26], we analyzed their sequence context. Although the chi-square test did no show significant differences compared to the percentages found for conserved sites, of all U-to-C transitions, the 3’ neighbor nucleotide was U in 40% of cases, A in 26.7%, G in 20% and C in 13.3%, which tend to the sequence context compatible with ADAR-mediated editing of the viral RNA.

**Table 5:** Characterization of mutant spectrum found in fecal specimens.

Sample	Mutations^a^/Nucleotides Sequenced						Nucleotide Mutation Frequency^c^		Amino Acid Mutation Frequency^c^		S_N_^d^
		Ts^b^	Tv^b^	Nsyn^b^	Syn^b^	Indel^b^ or Stop	Minimum	Maximum	Minimum	Maximum	
B-1	102/2267100	6	0	4	2	0	2.65×10^−6^	4.50×10^−5^	5.29×10^−5^	6.22×10^−5^	0.02
C-1	37/573750	1	0	0	1	0	1,74×10^−6^	6,45×10^−5^	0	0	0.02
D-1	139/1990800	2	0	0	2	0	1,00×10^−6^	6,98×10^−5^	0	0	0.02
D-6	252/1643850	16	1	11	6	0	1,03×10^−5^	1,53×10^−4^	2.01×10^−5^	1.70×10^−4^	0.05
F-1	107/653850	5	0	1	4	0	7,65×10^−6^	1,64×10^−4^	1.84×10^−5^	2.66×10^−4^	0.05

^a^ Mutations are those that vary relative to the corresponding consensus sequence. Nucleotides sequenced are the total number of nucleotides sequenced.

^b^ The mutations are classified into: transitions (Ts), transversions (Tv), nonsynonymous (Nsyn), synonymous (Syn) and insertion/deletion (Indel or Stop).

^c^ The minimum nucleotide mutation frequency is the number of different mutations found divided by the total number of nucleotides sequenced. The maximum nucleotide mutation frequency is the total number of mutations found divided by the total number of nucleotides sequenced. The maximum amino acid mutation frequency is the total number of nonsynonymous mutations divided by the number of amino acids encoded in the sequence analyzed. Mutation frequencies are expressed as substitutions per nucleotide or amino acid substitutions per amino acid.

^d^ The Shannon entropy was calculated as S_N_ = [∑_i_ (p_i_ × ln p_i_)]/lnN, where p_i_ is the frequency of each sequence and N is the total number of sequences.

**Fig 4:**
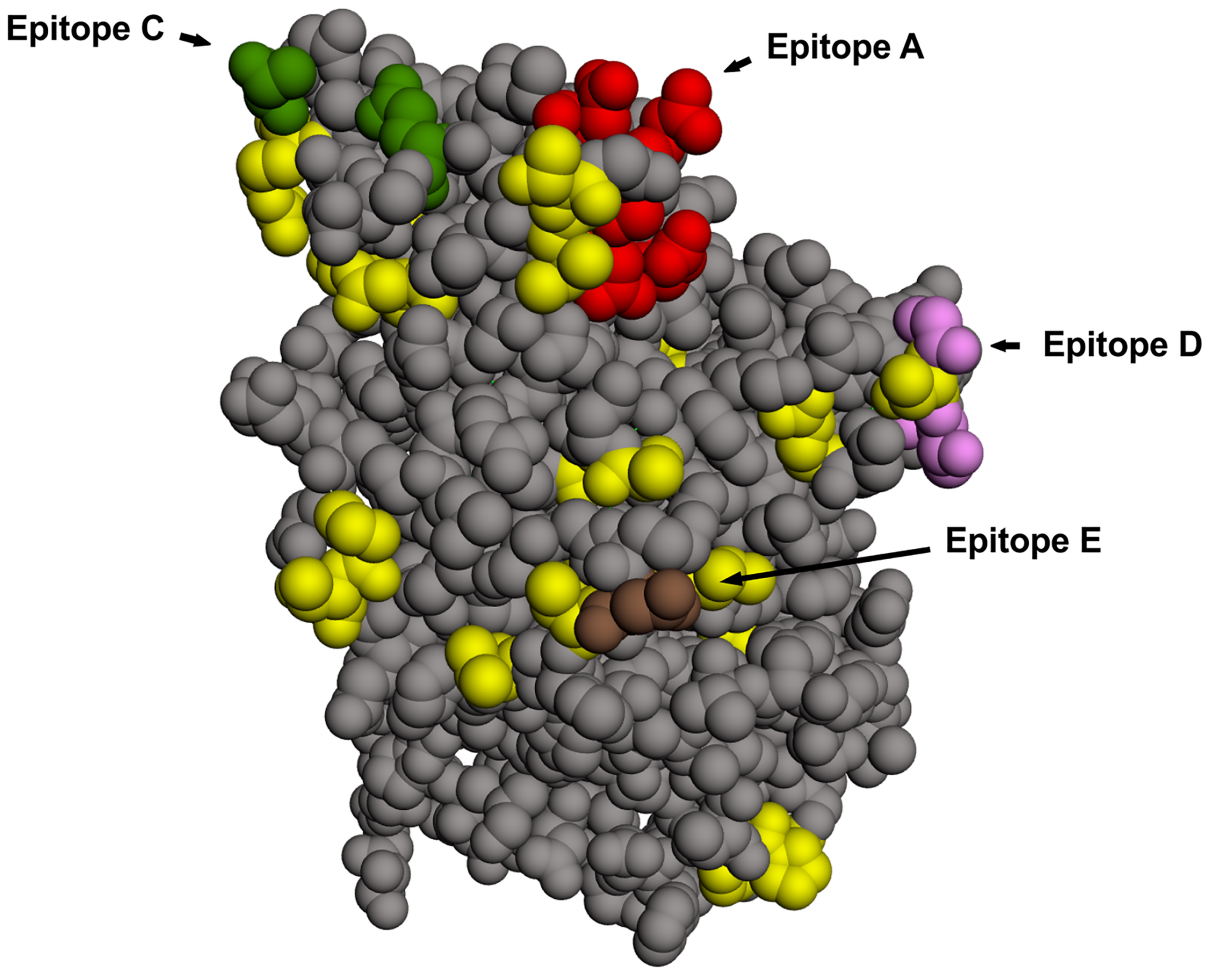
Amino acid variations in P domain. Amino acid changes observed in minor variants (yellow) were highlighted on the GII.4 P domain (side view), using the GII.4 2012 P2 domain structure (PDB id 4OOS; available at https://www.rcsb.org/structure/4OOS). The reference structure shows epitopes A (red), C (green), D (pink) and E (brown). The Jmol software was used to localize the amino acid residues (Jmol: an open-source Java viewer for chemical structures in 3D. http://www.jmol.org/).

At the amino acid level, the majority of the observed changes emerged at or near epitopes A, D and E, while some others were located at internal regions of the P dimer (Tables 3 and 4, and Fig 4) [14,24,25,27]. No changes were observed in HBGA binding pocket sites. Identified substitutions matched previously identified changes between different GII.4 variants (VP1 residues 289, 293, 294, 313, 314, 330, 339, 356, 358, 380, 394, 399, 404, 407, 413, 416, 426, 427, 433, 434, and 436). In two different individuals, mutations were detected at specific amino acids directly involved in epitope D (T394I) and epitope E (S407I) among the minor variants, suggesting that they may affect antibody recognition and HBGA binding.

## Discussion

In this work we used amplicon-NGS analysis to investigate the viral population of selected individuals linked to the same outbreak, as well as to analyze the intra-host HuNoV evolution dynamics over time. Since all investigated subjects suffered an acute infection, viral populations observed were highly homogeneous as expected. However, studied individuals who were asymptomatically infected had a higher haplotype diversity, suggesting that they may also act as a reservoir of novel genetic variants. Asymptomatically infected individuals are usually underreported and may transmit the virus in the absence of maximized hygiene habits.

Of note, the pattern of evolution of HuNoV variability over time differed in the 2 analyzed symptomatic subjects. While the single initial haplotype was maintained in one of them (subject A) during at least 8 days, diversity in subject D, which also was initially higher, increased from 3 to 18 haplotypes in only 5 days, revealing an average of 0.0062 nucleotide changes per nucleotide per day. Although still remarkable, this variation rate is significantly lower than what was described for immunocompromised patients chronically infected with HuNoV, which ranged between 0.03 to 0.37 nucleotide changes per day considering the full genome consensus sequence variation over time [16]. In addition to unknown individual factors, such as genetic differences or the history of previous infections by different strains of each subject, the degree of diversity in the transmitted/founding population that initiated the infection in both individuals may have also been different. Although our analysis did not indicate a correlation between haplotype diversity and viral load within a given sample, the initial viral load may be important in determining the evolution pattern. Individual D, who had the highest initial viral load at day 1 showed the highest diversity over time.

The effect of other factors related to the host on the degree of intra-host haplotype diversity could be especially observed in foodborne outbreak RCC11/10, which had been caused by a common source (contaminated shellfish). In this outbreak, a greater level of variability was found in the subject who did not develop symptoms. The relationship between the immune response raised to clear infection and the occurrence of symptoms is still poorly understood, and although a larger number of asymptomatic infected individuals should be tested to confirm this idea, our observations suggest that people infected by HuNoV who do not suffer symptoms may act as a reservoir for viral antigenic variants. While a properly functioning immune response may induce a higher fixation rate of mutations [28], it would be interesting to study whether symptomatology and/or virus severity may also affect it.

The NGS analysis of samples belonging to UVEVV51/10 outbreak occurring at a nursing home allowed us to confirm earlier results reported by Sanger sequencing [18]. While subjects C and D shared some of the haplotypes, sequences detected in subjects E and F were markedly different, suggesting more than one introduction of HuNoV in the nursing home. However, since transmission has been shown to be an important source of diversity and minor variants at frequencies as low as <0.01% have been shown to be efficiently transmitted from a donor to a recipient [12], viral transmission between subjects C or D and E and F cannot be completely ruled out. Quality filters performed during our NGS analysis excluded haplotypes at frequencies lower than 0.1%. Indeed, the type of transmission event (foodborne, fecal-oral, vomit-oral, etc) may also affect viral population diversity. Workers at nursing homes may be frequently exposed to episodes of vomiting and diarrhea and minor variants may disseminate more easily [29]. Indeed, the total number of different haplotypes found in the nursing home outbreak was higher than in the foodborne outbreak.

It is described that HuNoV GII.4 is able to generate new antigenic variants that have the potential to escape herd immunity by accumulation of mutations at major blockade epitopes [30,31,32,33]. In our study, numerous SNPs were found in sequence variants from both outbreaks, and 21 of them produced amino acid changes at sites previously determined to be functionally significant. Some of these changes occurred at or near described epitopes A, B, D, and E, while none of them affected any of the described receptor binding pocket sites.

In summary, NGS analysis is useful to characterize the quasispecies diversity and evolution over time, and may be a powerful tool to understand and confirm viral transmission between different hosts. Although evolution of viral population within an infected host may be subject-specific, it may also occur in asymptomatically infected individuals.
