# Correction: Characterization of intra- and inter-host norovirus P2 genetic variability in linked individuals by amplicon sequencing

**DOI:** 10.1371/journal.pone.0209714

**Published:** 2018-12-19

**Authors:** Aurora Sabrià, Rosa M. Pintó, Albert Bosch, Josep Quer, Damir Garcia-Cehic, Josep Gregori, Angela Dominguez, Mónica Carol, Maria-Rosa Sala-Farré, Susana Guix, the Working Group for the Study of Outbreaks of Acute Gastroenteritis in Catalonia

There are errors in the Funding section. The correct funding information is as follows: This work was supported in part by grants from the Fondo de Investigaciones Sanitarias Instituto de Salud Carlos III, Madrid, Spain [PS09/02516 (AD), PI 16/02005 (AD) and PI16/00337 (JQ]); https://portalfis.isciii.es] (co-funded by European Regional Development Fund “Investing in your future”); the Catalan Agency for the Management of Grants for University (AGAUR Grant Number 2017/SGR 1342); by the Spanish Ministry of Economy and Competitiveness (MINECO) [BIO2011-23461 (RMP); http://www.mineco.gob.es/]; by the Centro para el Desarrollo Tecnológico Industrial-CDTI from MINECO [IDI-20151125 (JQ); https://www.cdti.es/]; and by the XRB Biotechnology Reference Network (Generalitat de Catalunya) [(AB); http://www.xrb.cat]. The authors declare a commercial affiliation of JG with Roche Diagnostics S.L. which only provided support in the form of JG’s salary, but did not have any additional role in the study design, data collection and analysis, decision to publish, or preparation of the manuscript. The specific roles of these authors are articulated in the ‘author contribution’ section.
